# Prospective investigation of polyomavirus infection and the risk of adult glioma

**DOI:** 10.1038/s41598-021-89133-3

**Published:** 2021-05-05

**Authors:** Kathleen M. Egan, Youngchul Kim, Noemi Bender, James M. Hodge, Anna E. Coghill, Stephanie A. Smith-Warner, Dana E. Rollison, Lauren R. Teras, Tom K. Grimsrud, Tim Waterboer

**Affiliations:** 1 Department of Cancer Epidemiology, H. Lee Moffitt Cancer Center and Research Institute, 12902 Magnolia Drive, Tampa, FL 33612 USA; 2 Department of Biostatistics, H. Lee Moffitt Cancer Center and Research Institute, Tampa, FL 33612 USA; 3 Infections and Cancer Epidemiology, German Cancer Research Center (Deutsches Krebsforschungszentrum, DKFZ), 69120 Heidelberg, Germany; 4 Department of Population Science, American Cancer Society, Atlanta, GA 30303 USA; 5 Departments of Nutrition and Epidemiology, Harvard T.H. Chan School of Public Health, Boston, MA 02115 USA; 6 Department of Research, Cancer Registry of Norway, 0379 Oslo, Norway

**Keywords:** Cancer, Cancer epidemiology, Cancer prevention, CNS cancer

## Abstract

Glioma is an aggressive primary tumor of the brain with a poorly understood etiology. We studied the association of 4 human polyomaviruses (HPyV)—JC virus (JCV), BK virus (BKV), human polyomavirus 6 (HPyV6), and Merkel cell polyomavirus (MCPyV) with glioma risk within the Cancer Prevention Study II in the US (CPS-II) and the Janus Serum Bank in Norway. Cohort participants subsequently diagnosed with glioma from the CPS-II (n = 37) and Janus Serum Bank (n = 323), a median of 6.9 and 15.4 years after blood collection, respectively, were matched to individual controls on age, sex, and date of blood draw. Serum antibodies to the major viral capsid protein (VP1) were used to establish infection history for each polyomavirus. Odds ratios (ORs) and 95% confidence intervals (CIs) were estimated using conditional logistic regression. In the Janus Serum Bank, MCPyV infection was associated with a higher risk of glioma overall (OR: 1.56; 95% CI 1.10, 2.19). A modest, nonsignificant positive association with MCPyV infection was also observed in CPS-II (OR: 1.29; 95% CI 0.54, 3.08). In both cohorts, glioma risk was not significantly related to infection with JCV, BKV or HPyV6. The present study suggests that MCPyV infection may increase glioma risk.

## Introduction

Malignant glioma is an aggressive primary neoplasm of the brain with poorly understood etiology^1^. Risk factors include advancing age, male gender, European ancestry^2^, inherited genetic factors^3,4^, taller height^5^, and an older age at menarche in women^6^. Ionizing radiation is the only well-validated environmental risk factor^7^. A reduced incidence of glioma is observed in persons with atopy^8,9^ or with elevated prediagnostic levels of the immune biomarker IgE^10–12^, suggesting a role for immune function in glioma and consistent with a potential role for infection in the onset of these tumors^13^. A wide range of infections cross the blood–brain-barrier^14^ and are suspected to play a role in neuroinflammation and may contribute to neurodegenerative disease^15^ and neurological brain tumors^16^. While varicella zoster virus (VZV), a herpes virus that causes chicken pox and shingles, has consistently been linked to a *reduced* risk of glioma^17–19^, epidemiologic research on the role of other viruses in glioma remains limited.

Polyomaviruses (PyVs) are small, non-enveloped DNA viruses that exhibit the capacity to mediate cell transformation and tumorigenesis in different model systems^20^. A total of 14 PyVs are known to infect humans (human PyV, HpyV). Several of the HPyVs are neurotropic and/or have been linked to cancer in humans or other animals^21,22^. JC virus (JCV)^23^ and BK virus (BKV)^24^ have been postulated to play a role in brain tumors^25^. JCV is the cause of progressive multifocal leukoencephalopathy^26^, a fatal demyelinating disease of the central nervous system. BKV is the causal agent in polyomavirus-associated nephropathy that occurs in patients undergoing immunosuppressive therapy. Both viruses are highly oncogenic when injected into the brain of experimental animals^25^. Merkel cell polyomavirus (MCPyV)^27^, is the only known oncogenic PyV in humans and is the postulated cause of Merkel cell carcinomas (MCC) of the skin^28^. A raccoon PyV (RacPyV) closely related phylogenetically to MCPyV has been found to cause glioma-like tumors in raccoons^29^. The International Agency for Research on Cancer classifies Merkel cell polyomavirus (MCPyV) as a probable carcinogen whereas BKV and JCV are classified as possible carcinogens^30^ based on “sufficient evidence in experimental animals” but “inadequate evidence of carcinogenicity in humans”.

The role of polyomavirus infection in relation to glioma risk in humans is unknown. In the only prospective study to date^31^, antibodies to JCV, BKV, and simian virus 40 (SV40) measured in serum collected 1–22 years before glioma diagnosis were evaluated for association with incident glioma. Glioma cases (n = 44) and age-, gender- and race-matched controls (n = 88) were identified from participants of two specimen banks in Washington County, Maryland. The study detected no association with SV40. A nonsignificantly positive association was found for JCV (OR: 1.46), and an inverse association was found for BKV (OR: 0.66), with suggestively stronger but nonsignificant associations reported when restricting to grade IV glioblastomas (GBM) (ORs of 2.38 and 0.53, respectively).

Using a nested case–control design within two prospective cohort studies with biobanked collected blood, the Janus Serum Bank and the Cancer Prevention Study II (CPS-II) Nutrition cohort, we conducted an exploratory investigation of 4 polyomaviruses, JCV, BKV, HPyV6 and MCPyV, in relation to glioma risk. A multiplex assay was used to detect serum antibodies to the major capsid proteins (VP1) of each virus. To avoid potential bias in results from effects of preclinical disease on serum antibody titers, the study was restricted to cases with blood collected a minimum of 3 years (in the CPS-II) or 5 years (in the Janus Serum Bank) prior to glioma diagnosis.

## Methods

### Study populations

Data from two cohorts were included in the present study: (1) the Cancer Prevention Study-II (CPS-II) Nutrition cohort, a US prospective study^32^; and (2) the Janus Serum Bank, a population-based prospective study based in Norway^33^. Baseline characteristics of participants from each cohort are shown in Table 1. Incident primary intracranial glioma cases (ICD9 and 10 topography codes: 191 and C71, respectively) were comprised of WHO grade IV glioblastomas (GBM) (ICD-O-3 histology code: 9440-9441), and lower grade gliomas (‘nonGBM’)(ICD-O-3 histology codes: 9380, 9382, 9400-01, 9410-11, 9420, 9424-25, 9450-9451)^34–36^. In CPS-II, among the 32,609 cancer-free participants that provided a blood sample between 1998 and 2001 who were followed through the end of 2013, 37 glioma cases diagnosed a minimum of 3 years after sample collection were included in the present study. For each case, we randomly selected two controls from participants who provided a blood sample, and were alive and had no personal history of cancer (excluding basal and squamous cell carcinoma) as of the diagnosis date of the case. Each control was individually matched to its case on birth date within 1 year, sex, and date of blood draw within 6 months.

**Table 1:** Descriptive characteristics of nested case–control studies of Polyomaviruses and glioma risk: Cancer Prevention Study II and Janus Serum Bank.

	Cancer Prevention Study II				Janus Serum Bank					
	All		GBM		All		GBM		nonGBM	
	Cases	Controls	Cases	Controls	Cases	Controls	Cases	Controls	Cases	Controls
All	37	74	27	54	323	323	196	196	127	127
Male	17 (46%)	34 (46%)	13 (48%)	26 (48%)	219 (67%)	219 (67%)	141 (72%)	141 (72%)	78 (61%)	78 (61%)
Median age at blood draw (range)	71 (59–77)	71 (59–78)	71 (59–77)	71 (59–78)	41 (19–67)	41 (19–68)	41 (19–67)	41 (19–68)	41 (19–67)	41 (19–66)
Median year of blood collection (range)	2000 (1999–2001)	2000 (1999–2001)	2000 (1998–2001)	2000 (1998–2001)	1986 (1972–1991)	1986 (1972–1991)	1986 (1972–1991)	1986 (1972–1991)	1986 (1972–1991)	1986 (1972–1991)
Median years from blood draw to glioma diagnosis (range)	6.9 (3.4–13.0)	na	6.8 (3.4–11.5)	Na	15.4 (5.1–34.9)	na	16.4 (5.1–35)	na	14.6 (5.1, 35.0)	na

In the Janus Serum Bank, glioma cases and controls were identified from a previous nested case–control study of glioma^12^; a total of 323 glioma cases diagnosed a minimum of 5 years after blood collection were included in the present analysis. Each control was individually matched to its case on birth date within 2 years, sex, date of blood draw within 12 months, and county of residence, and was alive and free from any cancer except basal and squamous cell carcinoma on the date of glioma diagnosis of the case subject. All subjects were of European ancestry.

All research was performed in accordance with the human subjects’ protection principles (Declaration of Helsinki), and the study was approved by the ethical review board of the Moffitt Cancer Center and from both participating cohorts (CPSII-NC: Emory University IRB #00045780; Janus: Regional Committees for Medical and Health Research Ethics, Application 9821, #2017/2140/ REK-s-o-B). All participants of the CPSII-NC provided written informed consent at the time of blood draw. As described previously^37^, Janus Serum Bank donors gave broad verbal consent (1973–1992) or provided explicit written informed consent (1997–2004) for use of samples for cancer research, in accordance with Norwegian legal requirements over time. The Norwegian Data Protection Authority has approved use of Janus data and samples collected between 1972 and 2004, while requiring that donors are free to unconditionally withdraw their consent at any time. All research projects using Janus specimens and data from the Cancer Registry of Norway also need approval from a Regional Committee for Medical and Health Research Ethics.

### Multiplex serological assays

Multiplex serological analyses to measure antibodies to the viral proteins were conducted at the German Cancer Research Center (Deutsches Krebsforschungszentrum, DKFZ) in Heidelberg, Germany. This method allows the simultaneous measurement of antibody responses to multiple proteins in biologic samples^38^. Polyomaviruses lead to strong and reproducible antibody responses to the major capsid protein VP1^39,40^, a sensitive cumulative marker of past or present infection. For the current study, a prediagnostic plasma sample was obtained from each case and control from both participating cohort studies and shipped to the lab in Heidelberg for the analysis of serological markers of infection. Serological analyses were performed by fluorescent bead-based multiplex serology as previously described^38–41^. Briefly, the method is based on a glutathione S-transferase (GST) capture assay combined with fluorescent-bead (Luminex) technology. Proteins of interest are fused with an N-terminal GST domain. These recombinant GST-VP1 fusion proteins are affinity-purified in situ through binding to glutathione casein-coated fluorescence labelled polystyrene beads. Each fusion protein is bound to a spectrally distinct bead set (SeroMap, Luminex). The bead mix is then incubated in 96-well plates with participant plasma. Bound antibodies were detected with biotinylated triple (IgG, IgA, IgM) goat anti-human secondary antibody and streptavidin-R phycoerythrin reporter conjugate. A Luminex analyzer was used to identify the internal color of the individual beads and to quantify their reporter fluorescence (expressed as median fluorescence intensity (MFI)) as a measure of viral antibody titer. GST only (i.e. without viral antigen fusion component) served for background determination (i.e., negative control). Auto-fluorescence of each bead set and background reactions resulting from binding of secondary reagents were determined in one well per plate without human serum. Mean background values were subtracted from raw antibody output to determine antigen-specific reactivity (i.e., unit of statistical analysis). The serological analyses included the viral major capsid protein 1 (VP1) of four human polyomaviruses (JCV, BKV, HPyV6 and MCPyV). All samples from both cohorts were analyzed in a single laboratory batch avoiding measurement error otherwise introduced by the need for a bridging panel to normalize results across studies. Laboratory personnel were blinded to case–control status.

### Statistical analysis

Odds Ratios (OR) and 95% Confidence Intervals (CI) for associations of PyV infection and glioma risk were calculated using conditional logistic regression. All models were inherently adjusted for matching factors. We considered the association of glioma with seroprevalence (i.e., the presence or absence of VP1 antibodies) of each of the four HPyVs, with seropositivity for each HPyV defined as virus-specific VP1 antibody level ≥ 250 MFI^41^. In Janus, associations of glioma with viral markers were considered overall, and according to glioma grade including WHO grade IV GBM (n = 196) and all lower glioma grades, combined (n = 127) including 56 astrocytomas (ICD-O-3: 9400-01, 9410-11, 9420, 9424-25), 27 oligodendrogliomas (9450 and 9451), 24 astrocytic tumors (9380) and 20 mixed gliomas (9382). (Data were too sparse to examine associations with individual lower grade tumors in Janus, and with nonGBMs (n = 10 cases) in CPS-II.) In the Janus Serum Bank (in which larger numbers permitted an evaluation of dose response), we examined associations according to increasing MFI as a measure of viral burden. Tertiles of MFI among seropositive subjects were compared to a seronegative referent (i.e., < 250 MFI), with cutpoints established in seropositive controls. For JCV, tertile boundaries were 253–598, 601–1352, and 1365–14,967 for tertiles 1, 2, and 3, respectively; these figures were 251–1095, 1111–3695, and 3703–18,864, respectively, for BKV; 255–3052, 3069–5769, and 5774–18,637, respectively, for HPyV6; and 251–2837, 2846–6327, and 6346–17,802, respectively, for MCPyV.

### Disclaimer

The views expressed here are those of the authors and do not necessarily represent the views of the American Cancer Society or the American Cancer Society—Cancer Action Network.

## Results

The analyses were based on 37 glioma cases (27 GBMs) and 74 controls from CPS-II and 323 glioma cases (196 GBMs) and 323 controls from the Janus Serum Bank (Table 1).

In the CPS-II, approximately half of the cases and controls were male (46%) and the median age at blood collection was 71 years (range 59–77 years). Year of blood collection ranged from 1999 to 2001 (median: 2000). Glioma cases were diagnosed between 2002 and 2013. The median interval between blood collection and glioma diagnosis was 6.9 years and ranged from 3.4 years to 13.0 years. In the Janus Serum Bank, a majority of the cases were male (67%) and the median age at blood collection was 41 years (range 19–67 years), with a tight distribution on age (interquartile range in age: 39.8–42.6 years). Year of blood draw ranged from 1972 to 1991 (median: 1983). Glioma cases were diagnosed between 1980 and 2009. The median interval between blood collection and glioma diagnosis was 15.4 years and ranged from 5.1 to 34.9 years. In both studies, controls were similar to cases on age at blood draw, gender, and year of blood draw as these were matching factors. In CPS-II, the median age and interquartile range (IQR) at glioma diagnosis was 79 (75–81) and 76 (73–78), in males and females, respectively. In the Janus Serum Bank, in which participants were younger at enrollment, the median age and IQR of cases at glioma diagnosis for males and females were 57.1 (50.4–63.3) and 56.3 (52.1–60.3), respectively.

Seroprevalence of antibody markers among controls was 51% and 57% for JCV; 64% and 62% for MCPyV; 83% and 78% for BKV; and 77% and 80% for HPyV6, in the CPS-II and Janus Serum Bank, respectively, similar to seroprevalences reported for these PyVs in immunocompetent populations from different continents^21,41–46^.

In the Janus Serum Bank, infection with MCPyV was associated with a significantly increased risk of all gliomas combined (OR: 1.56; 95% CI 1.10–2.19) (Table 2) and for overall glioma risk in men (OR: 1.59; 95% CI 1.03–2.44) (not shown) ); positive associations of similar magnitude were observed for GBM (OR: 1.53; 95% CI 0.99–2.36) and nonGBM (OR: 1.60; 95% CI 0.92–2.8), and for overall glioma risk in women (OR: 1.50; 95% CI 0.85–2.64) (not shown), although none were statistically significant due to more limited case numbers. A modest and non-significant positive association with MCPyV infection was also observed in CPS-II (Table 2) for all gliomas combined (OR: 1.29; 95% CI 0.54–3.08).

**Table 2:** Association of polyomavirus seroprevalence with glioma risk in the Cancer Prevention Study II and Janus Serum Bank.

Virus	Cancer Prevention Study II				Janus Serum Bank			
	Cases seropositive/seronegative	Controls seropositive/seronegative	OR	95% CI	Cases seropositive/seronegative	Controls seropositive/seronegative	OR	95% CI
BKV	31/6	61/13	1.09	0.39–3.03	259/64	250/73	1.18	0.81–1.73
JCV	16/21	41/33	0.57	0.24–1.36	166/157	182/141	0.82	0.60–1.12
HPyV6	28/9	57/17	0.93	0.39–2.25	258/65	259/64	0.98	0.67–1.44
MCPyV	25/12	46/28	1.29	0.54–3.08	231/92	201/122	1.56	1.10–2.19

Analyses based on 37 glioma cases (27 GBMs) and 74 matched controls for CPS-II and 323 glioma cases (196 GBMs and 127 nonGBMs) and 323 matched controls in Janus Serum Bank. Referent group in all analyses comprised of seronegative (median fluorescence intensity < 250) individuals. ORs and CIs were conditioned on the following matching factors: birth date within 1 year, sex, and date of blood draw within 6 months for CPSII; and age within 2 years, sex, county of residence, and date of blood collection within 12 months for Janus.

*BKV* BK virus, *JCV* JC virus, *HpyV6* Human Polyomavirus 6, *MCPyV* Merkel Cell Polyomavirus, *GBM* glioblastoma, *OR* odds ratio, *CI* confidence interval.

In the CPS-II and Janus Serum Bank, the risk for all gliomas combined was not significantly related to seroprevalence of BKV (ORs: 1.09 (95% CI 0.39–3.03) and 1.18 (95% CI 0.81–1.73), respectively), JCV (ORs: 0.57 (95% CI 0.24–1.36) and 0.82 (95% CI 0.60–1.12), respectively), or HPyV6 (ORs: 0.93 (95% CI 0.39–2.25) and 0.98 (95% CI 0.67–1.44), respectively) (Table 2). Results for the 3 viral markers were similarly nonsignificant in both cohorts when restricting to GBM, and in the Janus Serum Bank when restricting to nonGBM (not shown). (Data were too sparse in CPS-II to evaluate associations for nonGBM, n = 10 cases.) All results for these 3 PyVs were similarly nonsignificant when considered separately in males and females (not shown).

We examined cross-reactivity between the 4 examined PyVs^41^ in the Janus Serum Bank and found modest correlations in antibody titers ranging from r = 0.082 (*p*-value = 0.035) for the correlation of BKV with HPyV6, to r = 0.167 (*p*-value < 0.001) for the correlation of MCPyV with HPyV6. Therefore, in the Janus Serum Bank we examined associations of glioma with seroprevalence of each PyV adjusted for seroprevalence of the other PyVs and all results were essentially unchanged (not shown). For example, the odds ratio for MCPyV infection was 1.56 (95% CI 1.10–2.19) in the unadjusted model and 1.61 (95% CI 1.13–2.28) in the model adjusted for seroprevalence of BKV, JCV and HPyV6.

When examining risk according to tertiles of antibody levels to BKV, JCV, and HPyV6 in the Janus Serum Bank, no significant trends in risk emerged for all gliomas combined (*p*-value trends = 0.761, 0.579 and 0.798, respectively) (Table 3), or among GBMs and nonGBMs when considered separately (data not shown). For MCPyV, a positive association of borderline significance was observed with increasing titer in gliomas overall (*p*-value trend = 0.052) (Table 3). The result for MCPyV differed according to glioma subtype (Supplemental Table S1): for GBM, ORs rose with increasing tertile (OR for tertile 3 vs seronegativity: 1.80 (95% CI 1.03–3.15) with a significant trend (*p*-value trend = 0.048), whereas for nonGBM, the OR was significantly elevated only in the first tertile (OR for tertile 1 vs seronegativity: 2.11 (95% CI 1.06–4.19) and declined with increasing tertile (OR for tertile 3 vs seronegativity:1.29 (95% CI 0.59–2.81) with a nonsignificant trend (*p*-value trend = 0.575).

**Table 3:** Association of Polyomavirus seroprevalence and antibody titer with glioma risk in the Janus Serum Bank.

Virus	Median Fluorescence Intensity of PyV										
	Referent	Tertile1			Tertile2			Tertile3			p.trend
	MFI	mMFI	OR	CI	mMFI	OR	CI	mMFI	OR	CI	
BKV	< 250	596	1.19	0.77–1.85	2,239	1.33	0.82–2.14	6,222	1.07	0.67–1.70	0.761
JCV	< 250	388	0.73	0.48–1.13	914	0.79	0.52–1.22	2,361	0.96	0.61–1.49	0.579
HPyV6	< 250	1,389	1.11	0.68–1.82	4,476	0.87	0.55–1.37	8,118	1.01	0.64–1.58	0.798
MCPyV	< 250	1,158	1.73	1.12–2.68	4,814	1.34	0.86–2.07	8,596	1.63	1.04–2.56	0.052

Referent group in all analyses is comprised of seronegative individuals, i.e., MFI < 250. Median MFI in reference group is 110.5 for BKV; 81.0 for JCV; 9.0 for HPyV6; and 39.0 for MCPyV. Tertile cutpoints in the JANUS Serum Bank established among seropositive individuals. ORs and CIs were conditioned on the following matching factors: 2-year age interval, sex, county of residence, and date of blood collection within 12 months.

*BKV* BK virus, *JCV* JC virus, *HpyV6* Human Polyomavirus 6, *MCPyV* Merkel Cell Polyomavirus, *PyV* polyoma virus, *mMFI* median value of the median fluorescence intensity in each tertile, *OR* odds ratio, *CI* confidence interval.

For MCPyV, ORs for all gliomas combined were more elevated with increasing length of time between blood collection and glioma diagnosis (ORs: 1.35 (95% CI 0.76–2.41), 1.61 (95% CI 0.89–2.90), and 1.75 (95% CI 0.95–3.23), for ≤ 12.6 years, > 12.6 to < 18.5 years, and ≥ 18.5 years, respectively) and age at blood collection (ORs: 1.44 (95% CI 0.76–2.72), 1.48 (95% CI 0.94–2.34), and 2.14 (95% CI 0.87–5.26), for ≤ 40 years, 41–45 years, and > 45 years, respectively), though no individual OR was statistically significant. Infection with JCV, BKV and HPyV6 were unrelated to glioma risk regardless of elapsed time between blood collection and glioma diagnosis and subject age at blood collection. In Janus, blood samples were collected over a 20 year period (between 1972 and 1991) and results were similar for samples collected in the first half (i.e.,1972–1981) versus the second half (i.e., 1982–1991) of the interval for each of the 4 HPyVs that we studied (Supplemental Table S2). For example, a positive association for MCPyV seroprevalence was demonstrated for the period from 1972 to 1981 (OR: 1.74; 95% CI 0.99, 3.05; p = 0.055) and for the period from 1982 to 1991 (OR: 1.46; 95% CI 0.95, 2.24; p = 0.086), though neither result was statistically significant in stratified analyses.

## Discussion

This is the first large prospective study to examine JCV, BKV, MCPyV and HPyV6 as potential causal agents in glioma. We found no consistent evidence supporting a role for BKV, JCV or HPyV6. In contrast, infection with MCPyV was positively associated with the risk of glioma. In Janus, the association with MCPyV was observed regardless of glioma subtype and gender. To our knowledge, this is the first study to implicate MCPyV infection in the development of a CNS tumor in humans, following evidence that a phylogenetically closely related virus (RacPyV) causes glioma-like tumors in raccoons^29,47^.

One previous prospective study^31^ based on a small set of incident cases (44 gliomas) examined glioma risk in relation to BKV and JCV infection. The authors reported a suggestive positive association with JCV and *inverse* association with BKV and findings that were more pronounced among GBMs (ORs of 2.38 and 0.53, respectively). The current study yielded nonsignificant results for both BKV and JCV, consistent with a lack of conclusive evidence of a role for these viruses in the development of human tumors^48^.

The present findings for MCPyV are of interest in view of the established association of this virus with Merkel cell carcinoma (MCC), an aggressive neuroendocrine tumor of the skin^28,49^. The etiology of MCC has been linked to ultraviolet light (UV) exposure and to infection with MCPyV. The attributable risk of each exposure depends on geography: in northern latitudes, the majority of MCC cases are of viral etiology, whereas in areas with high UV exposure, more cases are related to UV-mediated carcinogenesis^50^. UV exposure is thought to be involved in both viral-mediated and non-viral-mediated carcinogenesis by contributing to immunosuppression or DNA damage, respectively. With the exception of our report, to our knowledge no other tumor in humans has been linked to MCPyV exposure.

A biologic mechanism linking MCPyV infection to the risk of glioma is unknown. Whereas MCPyV is not specifically known to cross the blood–brain-barrier^14^, other human PyVs such as JCV are known to do so^26^, as does RacPyV^29^. In MCC, viral DNA is found integrated within the tumor genome in a clonal pattern suggesting that MCPyV infection and integration precede clonal expansion of tumor cells^27^. Whereas studies to date have not demonstrated MCPyV viral protein expression in human glial tumors^51,52^, MCPyV may contribute to glioma via a range of mechanisms^16^ such as neuroinflammation^53,54^, insertional mutagenesis^55–57^, or effects on gene expression^58^ that would not leave traces of viral integration in the tumor genome. Of note, RacPyV is considered the causal agent of neuroglial olfactory tract tumors in free-ranging raccoons^29^; the virus has not been found integrated in tumor DNA, suggesting a mechanism of PyV-mediated oncogenesis not requiring direct host cell integration^47^. MCPyV is a latent infection that occurs most often in early childhood^21,42^ and lies dormant but may reactivate in the context of immunosuppression to cause disease (i.e. BKV-related nephropathy in transplant patients and JCV-related PML in AIDS patients); therefore, IgG capsid antibodies reflect infection occurring decades prior to blood collection. The present association of MCPyV with glioma, including the suggestion of increased risk with advancing age at blood collection in the Janus series, is consistent with reactivation of latent virus possibly triggered by immune senescence with advancing age^59,60^ contributing to glioma onset. Why a common exposure like MCPyV might lead to the development of glioma is unknown. However, it is notable that MCC is also a rare tumor caused by exposure to this virus. Furthermore, a similar pattern is observed for Epstein-Barr virus, a ubiquitous Herpesvirus that is considered a necessary cause of nasopharyngeal carcinoma and Burkitt lymphoma, which are both rare cancers^61^. The association between EBV and rare malignancies suggests that as-yet unknown co-factors influence host susceptibility to this virus^62^. Analogously, MCPyV may contribute to neuro-oncogenesis only in the presence of host immune or genetic co-factors, a hypothesis that should be examined in future studies.

A key strength of the present study was the prospective design and investigation of prediagnostic blood samples for detection of viral antibodies. In the Janus Serum Bank, glioma cases were diagnosed over a broad interval following blood collection (5–35 years, with a median of 15 years) allowing for examination of potential latency of viral exposures on glioma risk. Furthermore, all analytic cases were diagnosed presumably outside of the preclinical window (3 or more years following blood collection in CPS-II, and 5 or more years in Janus) reducing potential influence of preclinical tumor on viral markers.

We acknowledge several limitations. Power to detect associations by gender and according to latency and antibody titer, were limited in the Janus Serum Bank and these evaluations could not be performed in CPS-II due to small numbers. For the same reason, we were unable to examine associations according to specific lower grade glioma histologic subtypes in Janus. Furthermore, IDH1 mutation status, which is an important molecular classifier of low grade gliomas and secondary GBM^63^, was not available in either study to examine whether associations vary according to IDH1 status. Lack of a statistically significant association for MCPyV in CPS-II could be explained by the small number of incident glioma cases or potentially the older age at onset of glioma in CPS-II cases when compared to Janus cases, although data from Janus Serum Bank suggests if anything a stronger association of the virus with glioma in older persons. Blood samples were collected in some cases many years before serologic testing for HPyV antibodies and long storage times could potentially have affected assay results. However, we note that antibodies are considered robust biomolecules^64^ that are stable with prolonged storage periods at low temperatures (at or below − 20 °C)^65^. In our study, we were able to detect a range of values for all HPyV antibodies, suggesting that the titer distribution at the time of measurement reflected the titer range at blood draw^40^. Furthermore, in studies of HPV^66^, antibody titers have been shown to be comparable in serum samples from Janus versus other cohorts with shorter storage times; as HPV antibody titers after natural infection are much lower than for HPyV (and therefore less robust in serological assays), these results provide further assurance that long storage times did not affect the present results. We lacked information on lifestyle factors such as smoking and obesity which are known to impact immune surveillance^67–72^ and may confound or alter strength of associations between viruses and cancer. Immunity is known to wane with age^59,73^ and we had a limited ability to consider associations according to age given the tight age distribution in the Janus Serum Bank. We also could not investigate associations by race/ethnicity. The study was based on a single blood measurement and infections that occurred after blood collection would have introduced misclassification of exposure; however, as studies have shown that seroprevalence of PyVs within individuals is stable over a decade or more in adults^40^, and that most individuals are infected with PyVs in childhood or adolescence^21,42^, we expect that misclassification of PyV infection status was limited among the middle aged adults in these cohorts. Finally, we did not consider antibodies to the viral T-antigens in MCPyV which are the key oncogenic drivers for MCC^74^; demonstation of a more prominent association of the T-antigen in MCPyV with glioma risk, as has been demonstrated in MCC^74^, would strengthen the case for a causal association.

In summary, in this pilot investigation we found the first evidence linking glioma risk specifically to infection with MCPyV but not with the 3 other PyVs examined. Investigations based on larger, diverse study populations with long-standing biorepositories are needed to clarify the role for MCPyV and other PyVs that infect humans^21,43^ as potential causal agents in glioma.

## Supplementary Information

Supplementary Information

## Data Availability

For access to data from the Janus Serum Bank approval must be obtained from The Regional Ethics Committee of Southern Norway and from the Cancer Registry of Norway (in charge of the Janus Serum Bank), according to national and European regulations of data protection. Data underlying the CPSII-NC results are available upon request by following the procedures outlined here: https://www.cancer.org/content/dam/cancer-org/research/epidemiology/cancer-prevention-study-data-access-policies.pdf
