# Supplementary material for: Prospective investigation of polyomavirus infection and the risk of adult glioma
Source: Sci Rep. 2021 May 5;11:9642. doi: 10.1038/s41598-021-89133-3 (PMC8100283; doi:10.1038/s41598-021-89133-3)
Supplement: Supplementary file 1 — Supplementary Information [file 41598_2021_89133_MOESM1_ESM.pdf]

Prospective Investigation of Polyomavirus Infection and the Risk of Adult Glioma

Kathleen M. Egan[1*]
Youngchul Kim [2]
Noemi Bender[3]
James M. Hodge[4]
Anna E. Coghill[1]
Stephanie A. Smith-Warner[5]
Dana E. Rollison[1]
Lauren R. Teras[4]
Tom K. Grimsrud[6]
Tim Waterboer

Supplemental Table 1. Association of MCPyV antibody titer with glioma risk according to glioma histology in the Janus Serum Bank

	Median Fluorescence Intensity of PyV														
	Seronegative		Tertile1				Tertile2				Tertile2				
	controls	cases	controls	cases	OR	CI	controls	cases	OR	CI	controls	cases	OR	CI	p.trend
ALL	122	92	63	81	1.73	1.12-2.68	72	72	1.34	0.86-2.07	66	78	1.63	1.04-2.56	0.052
GBM	73	55	40	44	1.48	0.83-2.61	43	44	1.36	0.78-2.37	40	53	1.80	1.03-3.15	0.048
nonGBM	49	37	23	37	2.11	1.06-4.19	29	28	1.27	0.63-2.59	26	25	1.29	0.59-2.81	0.575

PyV=polyoma virus; GBM= glioblastoma; OR=odds ratio; CI=confidence interval. Referent group in all analyses comprised of seronegative individuals, i.e., Median Fluorescence Intensity (MFI) < 250. Tertile cutpoints in the JANUS Serum Bank established among seropositive individuals. ORs and CIs were conditioned on the following matching factors: 2-year age interval, sex, county of residence, and date of blood collection within 12 months.

Supplemental Table 2. Association of Polyomavirus seroprevalence with glioma risk in the Janus Serum Bank according to year of blood collection

	Year of Blood Collection				
		**1972 to 1981**		**1982 to 1991**	
	Referent	134 cases and 134 controls		189 cases and 189 controls	
Virus	MFI	OR	CI	OR	CI
BKV	<250	1.17	0.68-2.01	1.20	0.71-2.04
JCV	<250	0.86	0.53-1.40	0.79	0.52-1.19
HPyV6	<250	1.42	0.68-2.97	0.85	0.54-1.34
MCPyV	<250	1.74	0.99-3.05	1.46	0.95-2.24

PyV=polyoma virus; MFI= median value of the median fluorescence intensity in each tertile; OR=odds ratio; CI=confidence interval. Referent group in all analyses comprised of seronegative individuals, i.e., MFI < 250. ORs and CIs were conditioned on the following matching factors: 2-year age interval, sex, county of residence, and date of blood collection within 12 months.
